# The Role of Emotional Competences in Parents’ Vaccine Hesitancy

**DOI:** 10.3390/vaccines9030298

**Published:** 2021-03-22

**Authors:** Teresa Gavaruzzi, Marta Caserotti, Irene Leo, Alessandra Tasso, Leonardo Speri, Antonio Ferro, Elena Fretti, Anna Sannino, Enrico Rubaltelli, Lorella Lotto

**Affiliations:** 1 Department of Developmental Psychology and Socialization, University of Padova, 35131 Padova, Italy; marta.caserotti@unipd.it (M.C.); irene.leo@unipd.it (I.L.); enrico.rubaltelli@unipd.it (E.R.); lorella.lotto@unipd.it (L.L.); 2 Department of Humanities, University of Ferrara, 44121 Ferrara, Italy; alessandra.tasso@unife.it; 3 Department of Prevention, AULSS 9 Scaligera, 37122 Verona, Italy; leonardosperi@gmail.com (L.S.); elena.fretti@gmail.com (E.F.); anna.sannino81@gmail.com (A.S.); 4 Department of Prevention, APSS Trento, 38123 Trento, Italy; antonio.ferro@apss.tn.it

**Keywords:** vaccines, parents, decision making, attitude, emotions

## Abstract

The role of parents’ emotional competencies on vaccine hesitancy and decision making has been seldom examined. Two studies investigated the relationship between parents’ attitudes towards childhood vaccines and self-reported behavior (Study 1) and between parents’ emotional competence and attitudes towards vaccines (Study 2). In Study 1, predictors of temporal, partial, or complete vaccine refusal (having voluntarily postponed/forgone some/all vaccines) were examined in 2778 parents. In Study 2, psychological predictors of the attitude towards vaccines were examined in 593 parents, using the Profile of Emotional Competence and the valence of mental images spontaneously associated with the term “vaccine”. In Study 1, attitudes were aggregated in three independent factors (concerns about vaccine safety; diseases prevented by vaccines; and naturalistic views) that independently predicted vaccine refusal. In Study 2, a significant mediational analysis showed a positive indirect effect of intrapersonal emotional competences on attitudes towards vaccines, through mental images associated with the word “vaccine”. Parents’ intrapersonal emotional competences affected all dimensions of attitudes towards vaccines, suggesting that being able to manage, identify, and recognize one’s own emotions is central to vaccine acceptance. These findings suggest that intervention strategies, rather than stressing the pro-social benefits of vaccinating, should focus on aspects related to one’s own emotions.

## 1. Introduction

Cognition and emotion both contribute to the development and the change over time of everyday preferences, judgments, and attitudes [1]. The main aim of this paper is to understand the role of emotional factors on parents’ attitudes towards childhood vaccination. Vaccination is considered one of the most important achievements of public health in the history of medicine. It is estimated that immunization prevents 2–3 millions of deaths every year [2]; nonetheless, immunization rates are often far from optimal for several reasons, including vaccine hesitancy. Vaccine hesitancy has been defined as a “delay in acceptance or refusal of vaccines despite availability of vaccine services” [3] by the World Health Organization (WHO) Strategic Advisory Group of Experts (SAGE) on Vaccine Hesitancy (but see [4]), and can lead to outbreaks of vaccine-preventable diseases [5,6].

Vaccine uptake is obviously the most important outcome from a public health perspective, but even parents of fully vaccinated children may have concerns about vaccines. Indeed, they have been referred to as “hesitant compliers” [7]. In the more recent literature, parents’ attitudes and behaviors are seen as comprising a continuum with endpoints of no doubts about vaccinating and no doubts about not vaccinating; between these endpoints are vaccine hesitancy behaviors that encompass vaccinating while having some concerns, vaccinating while having many concerns, partially vaccinating, and delaying vaccinations [3,8]. Thus, hesitancy is now considered a complex and multi-dimensional phenomenon, varying across time, place, and vaccines [9], and which specifically refers to parents’ attitudes towards vaccines [4].

To fully understand vaccine hesitancy, some research has focused on the psychological factors associated with parents’ attitudes and beliefs. For example, in a research study carried out in 24 countries, Hornsey and colleagues showed that anti-vaccination attitudes are strictly associated with conspiratorial thoughts, low tolerance to restrictions to personal freedom (i.e., reactance), disgust for blood and needles, and scarce acceptance of societal control on individuals, which are conceived as antecedents of attitudes [10]. Similarly, it has been shown that Trump voters are more concerned about vaccines than other voters and that this attitude was reduced when conspiracy and conservatism were controlled for [11].

Less work has been dedicated to understanding how emotion influences vaccine hesitancy; the present work aims at filling this gap in the literature. Affect is a response that is directed to a specific object or option and is experienced as a conscious or unconscious feeling. It demarcates a stimulus as positive or negative, based on its specific goodness or badness (the so-called affect heuristic) [12]. In other contexts, affect has been found to be a key factor in shaping preferences and choices [13]. For example, affective labels may help individuals to make better decisions, especially when they are overwhelmed by too much information, or they are stressed by a recent cancer diagnosis [14]. It is plausible to hypothesize that positive or negative attitudes towards vaccines originate from affective reactions towards them. For example, a false association between vaccines and autism, even when counteracted by true information, may linger in memory due to a familiarity backfire effect and contribute to maintaining myths about vaccines [15]. Indeed, preliminary evidence suggests that vaccine conspiracy is associated with unpleasant emotions towards vaccination, which, in turn, are associated with parents’ vaccine refusal [16].

Furthermore, empirical studies not related to vaccination have demonstrated a strong relationship between affect, decision making, and imagery. Many of these studies used a word-association technique, in which participants provide a thought or image that comes to mind after receiving a target stimulus (for example, the word “vaccine”), and then rate it. This imagery method has been used to measure the affective meanings that influence people’s preferences for different cities and states [17], as well as their support or opposition to technologies such as nuclear power [18]. In this study, we used the word imagery technique since it makes it possible to individuate the affective components of attitudes towards vaccines, especially when the affective components are immediate and not explicit. For example, people may show negative attitudes towards vaccines as they overestimate the probability of rare negative side effects, as they do about other hazards, like terroristic attacks, whose mental images are very dreadful [19]. At the same time, since the positive effects of vaccines are delayed in time and not visible to the individual, it is possible that people underestimate them. 

Individuals’ emotional reactions have also been investigated within the domain of Emotional Intelligence (EI) or Emotional Competence (EC), which influences the most crucial spheres of life: psychological well-being, physical health, social relationships, and professional success [20]. In line with some authors, we will use the term Emotional Competence (EC) to refer to competences that can be taught and learned [20]. It has been shown by several studies that EC modulates risk perception and the estimates of future negative or positive events. For example, people with low EC showed a stronger relation between more negative emotions and the belief that future terrorist attacks would occur in their small town rather than in farther locations, with a greater tendency to see immigrants as threatening [21,22]. Furthermore, it was demonstrated that EC moderates the relation between knowledge and decision making related to food. Specifically, higher levels of EC, compared to lower levels, moderated the perceived knowledge about food, favoring better quality decision making as for calories intake [23].

While EC predicts a number of important outcomes, the distinction between interpersonal and intrapersonal dimensions of EC has been often overlooked. When applied to acceptance of pediatric vaccines, we see this distinction as particularly important, since vaccinating can be considered as both an individualistic and an altruistic choice. Individualistic because it regards the parents’ own children, and altruistic because children who cannot be vaccinated, due to special medical conditions, are protected by herd immunity, which develops when 90–95% of people are vaccinated [24]. Based on the literature, we should expect that people with high intrapersonal EC are less influenced by unconscious emotions, and thus are better able to weigh them and appreciate the extremely low likelihood of severe side effects. As a result, these people should have more positive attitudes towards vaccination. In a similar fashion, we should expect that people with high (vs. low) interpersonal EC have more positive attitudes towards vaccinations since they are likely to recognize the benefits of vaccines to other people and empathize with those who need to be protected. In the present study, we used the Profile of EC [20] since, by measuring separately intrapersonal and interpersonal emotional competence, it fits particularly well with the goals of the present work. To the best of our knowledge, no previous study has linked emotional competence with attitudes towards vaccines.

The present research was conducted in the Veneto Region in Italy, between December 2016 and May 2017, as part of a series of studies commenced in 2006 aimed at monitoring the suspension of vaccination obligations and improving the quality of services [25]. Veneto had not imposed mandatory vaccinations since 2008, whereas in the rest of Italy four vaccines were mandatory (Italy’s new vaccine mandates were introduced after the conclusion of this research). Study 1 aimed at confirming the relationship between attitudes towards vaccines and behavior found in previous studies (e.g., [26]). Study 2 aimed to explore the relationship between parents’ emotional competence, their associations to the word “vaccine” (measured using the imagery method), and their attitude towards vaccines. 

## 2. Study 1

### 2.1. Materials and Methods

#### 2.1.1. Participants

A convenience sample of 2778 parents or legal guardians (89% female; mean age = 40 ± 6.7 years, ranging from 20 to 81) from the local health district (Ulss 6 Euganea) completed the questionnaire, as part of a survey monitoring attitudes towards vaccines in the Veneto Region, where vaccines mandates were suspended [27]. Thirty-nine participants were excluded from the analysis because their youngest child had not reached the age to start vaccination, leaving 2739 participants. The work described has been carried out in accordance with The Code of Ethics of the World Medical Association (Declaration of Helsinki). Similar to previous studies [28,29], participation was voluntary and anonymous, and, as the study was not aimed at research or actions of clinical relevance, it was exempt from ethical approval as per Italian standards. Participants were informed of the study aim and could leave the questionnaire or confirm their consent by completing it.

#### 2.1.2. Sample Size Justification

For the factor analysis of the 26-item questionnaire, with 10 cases per item, we estimated to need at least 260 participants. Considering that vaccine refusal is less common than vaccine acceptance, we aimed to recruit 260 vaccine refusing participants. Estimates of parents exhibiting vaccine refusal vary widely depending on the context, including the vaccines considered and the age of the child [8,30]. The refusal of all vaccines, together with late and selective vaccination, are estimated to range from 4 to 31% [8]. We used a conservative estimate of 10% and estimated to need a sample of at least 2600 participants.

#### 2.1.3. Material and Procedure

Data were collected between December 2016 and April 2017. The questionnaire consisted of 26 items evaluating parents’ attitudes towards vaccines (see Supplementary Material), developed on the basis of the existing literature and a series of group discussions led with the computerized Nominal Group Technique [31], and later refined [27]. Parents rated their agreement with each item on a scale from 1 (“completely disagree”) to 5 (“completely agree”). Self-reported behavior was coded as vaccine-refusal when parents of children aged 3 months or older declared that the child had received no vaccine (complete refusal), the child received only some of the vaccines offered (partial refusal), some vaccines were delayed voluntarily (temporal refusal), or both the child received only some of the vaccines offered and at least some vaccines were delayed voluntarily (partial and temporal refusal). Finally, the following socio-demographic variables were collected: age and gender of the youngest child; number of siblings; parents’ age, highest education attained, work status, and citizenship; whether the family is mono-parent; a measure of the ease to get to the end of the month (an indirect measure of socio-economic status); and the parent who completed the questionnaire. Other data were collected (e.g., sources of information accessed by parents personally and online; quality of relationships with health professionals) but are not the focus of the present study.

#### 2.1.4. Statistical Analyses

Descriptive statistical analyses were summarized producing frequency tables for categorical variables and using means with standard deviations (SDs) for continuous variables. A confirmatory factor analysis (a statistical technique that allows to unveil underlying common dimensions of a series of items based on participants’ responses) was applied to items assessing parents’ attitudes towards vaccines (Eigenvalues > 1; varimax rotation). Simple correlations between the factors emerged from the factor analysis were calculated. Logistic regression models were used to test the association of parents’ attitudes scores with vaccine-refusal. Results are presented using Odds Ratios (ORs). Statistical significance was assumed at the 5% level. Statistical analyses were performed using SPSS version 20.0 statistical software (IBM Corporation, Armonk, NY, USA).

### 2.2. Results

#### 2.2.1. Factor Analysis

The factor analysis identified a three factors structure, with an overall variance explained of 49.2% (see Supplementary Material).

Factor 1—Vaccine safety: Common fears and concerns about vaccinations with a focus on side effects (α = 0.87; 24.8% of variance explained, 16 items). Example of items included: “Health professionals do not want to recognize the link between vaccinations and some illnesses (e.g., allergies, asthma, others)”, “I would never forgive myself if my child would suffer from a severe adverse event from a vaccine”, and “Vaccines are administered when children are too young, they should be started later”.

Factor 2—Diseases prevented: Concerns about the negative consequences of the diseases prevented by vaccines (α = 0.81; 14.7% of variance explained, 5 items). Example of items included: “It is important to vaccinate children because the diseases that are prevented can have very serious effects”, “I worry that my child could get the diseases if he/she is not vaccinated against them”, and “I would never forgive myself if my child would suffer severe consequences from diseases that I could have avoided through vaccinations.”

Factor 3—Naturalistic views: Extreme views against vaccines and beliefs in natural protection (α = 0.64; 9.6% of variance explained, 5 items). Example of items included: “Breastfeeding protects children against any infection and therefore it is not necessary to vaccinate them until they are breastfed”, “Following healthy lifestyles disease can be avoided with no need to vaccinate children”, and “The disease prevented by the vaccine is less dangerous than the vaccine itself”.

#### 2.2.2. Descriptive Statistics and Correlations

Two hundred twenty-seven parents (8.3%) were classified as exhibiting vaccine-refusal. In 3.4% of the cases, their youngest child did not receive any vaccine (complete refusal). In 2.3% of the cases, children only received some of the vaccines offered (partial refusal), in 1.0% of cases they received all vaccines offered but at least some vaccines were delayed voluntarily (temporal refusal), and in 1.6% of cases they received only some of the vaccines offered and at least some vaccines were delayed voluntarily (partial and temporal refusal). In the remainder of the paper, we will refer to these groups as “vaccine-refusing parents”. The remaining parents (91.7%) reported having fully vaccinated their youngest child with no voluntary delays (for additional information about participants’ characteristics, see Table 1).

Data showed that, compared to parents who fully vaccinated their children with no voluntary delays, vaccine-refusing parents reported higher scores on “Vaccine safety” and “Naturalistic views”, and lower scores on “Diseases prevented” (see Table 2). In other words, they exhibited more common fears and concerns about vaccinations, more extreme views against vaccines and beliefs in natural protection, and fewer concerns about the diseases prevented by vaccines.

For both groups, we found significant correlations between the scores of the three factors measuring attitudes towards vaccines. These correlations were higher for parents who exhibited vaccine-refusal compared to those whose children completed all vaccines. In particular, correlations between “Diseases prevented” by vaccines and the other two factors were lower for vaccine-accepting parents (*r* = −0.24 and *r* = −0.22) than for vaccine-refusing parents (*r* = −0.62 and *r* = −0.64).

#### 2.2.3. Regression Analysis on Vaccine-Refusing Behavior

At the univariate level, parents’ attitude scores were associated with vaccine-refusal (see Supplementary Material). Specifically, as single predictors, parents’ scores on “Vaccine safety” and “Diseases prevented” were similarly predictive of behavior, but in opposite directions: for each additional point on “Vaccine safety” (i.e., common fears and concerns about vaccinations), the odds of exhibiting vaccine-refusal increased by more than 9 times (OR = 9.35; 95% CI: 7.45, 11.75; *p* < 0.001). Conversely, for each additional point on “Diseases prevented” (i.e., concerns about the diseases prevented by vaccines), the odds of being vaccine-refusing decreased by 0.11 times (95% CI: 0.09, 0.14; *p* < 0.001). Higher scores on “Naturalistic views” (i.e., beliefs in natural protection and extreme views against vaccines) were also associated with higher odds of showing vaccine-refusal, with an odds ratio of 3.63 (95% CI: 3.04, 4.33; *p* < 0.001). 

At the multivariate level, when all the three factors detailing parents’ attitude towards vaccines were included in the same regression model, all the factors showed an independent and significant contribution in predicting vaccine-refusal (Vaccine safety: OR = 6.08; 95% CI: 4.52, 8.18; *p* < 0.001; Diseases prevented: OR = 0.21; 95% CI: 0.17, 0.28; *p* < 0.001; Naturalistic views: OR = 0.60; 95% CI: 0.44, 0.82; *p* = 0.002) and the model showed a good explanatory power (Nagelkerke R^2^ = 0.59). Results were similar when controlling for socio-demographic variables (see Supplementary Material).

### 2.3. Discussion

Study 1 confirmed previous literature that attitudes towards vaccines were strongly associated with vaccine-refusal. At the same time the results confirmed the variability in attitudes towards vaccines. For example, a recent systematic review showed that parents’ support of mandatory vaccination programs varied considerably, ranging from 53 to 97%, as reported by different studies [32]. In our study, attitudes towards vaccines clustered around three factors: concerns about vaccine safety, worries about the negative consequences of the diseases prevented, and naturalistic views about protection against diseases. These results confirm that vaccine hesitancy is even more nuanced than a simple continuum, reinforcing previous research conducted in Italy yielding to a similar characterization of vaccine attitudes [31,33,34,35].

Importantly, parents may have concerns about the safety of vaccines while being also extremely worried about the negative consequences of the diseases prevented by vaccines. However, the strength of these concerns varies substantially. Indeed, the average scores showed that for vaccine-refusing parents, concerns about vaccine safety outweighed concerns about the diseases prevented by vaccines (3.90 vs. 3.26, *t* (226) = 5.51, *p* < 0.001), whereas for parents who fully vaccinated their child concerns about vaccine safety were considerably lower than concerns about the diseases prevented by vaccines (2.35 vs. 4.70, *t* (2511) = −123.65, *p* < 0.001).

It is noteworthy that, although being concerned about vaccine safety and holding naturalistic views were both associated with more negative views about vaccines (and were both predictive of higher vaccine-refusing behavior), they are two distinct factors. This implies that they tap into different underlying values. Indeed, while many parents hold some concern about vaccine safety, only a minority has naturalistic views against vaccines. This is also in line with previous research carried out in Italy, showing that safety concerns are the main reason reported for refusing (38.1%) or stopping (42.4%) vaccination [36]. Not surprisingly, parents’ scores on vaccine safety and naturalistic views were highly predictive of vaccine-refusal. The present findings are also in line with the international literature (e.g., [26,37]).

As outlined in the introduction, previous studies on factors predicting vaccine acceptance have rarely considered how parents’ emotional competencies affect decision making. Study 2 aims at filling this gap, exploring the relationship between these competencies and attitude towards vaccines.

## 3. Study 2

### 3.1. Materials and Methods

#### 3.1.1. Participants

A convenience sample of 593 parents or guardians (83.3% female; mean age = 37 ± 5.8 years, ranging from 20 to 66 years) of children attending public nurseries and kindergartens completed the questionnaire in a confidential format. Six additional questionnaires were not included in the analyses as respondents indicated that they already participated in Study 1. The University of Padova ethical committee for psychological research approved the study (protocol number 17_2175). 

#### 3.1.2. Material and Procedure

To hand out the questionnaires, we partnered with the public day-care nursery schools and kindergartens in Padova, Italy. Between April and May 2017, teachers handed out to parents the questionnaire along with a letter describing the project and an informed consent form. Parents who decided to participate in the study took the questionnaire home and returned it to the schools, placing it in a closed box to avoid any risk of identifying their answers. Due to the nature of the study, no formal sample size calculation was performed.

The questionnaire included four different sections. In the first section, we asked respondents to complete an imagery task. On the top of the page, they were presented with the word “vaccine” and were asked to report the first three images or concepts that came to their minds when thinking about vaccines. Once the respondents reported the images that came to their minds, they were asked to rate them on a 5-point scale ranging from −2 (“absolutely negative”) to +2 (“absolutely positive”). Similar to previous studies using this paradigm, we were not interested in the mental images per se but in their ratings [17,18]. For the analyses, we averaged, for each participant, the ratings for the three images. In the second section, attitudes towards vaccines were measured asking respondents to report their agreement with the same 26 items used in Study 1 (see Supplementary Material). Since a factor analysis confirmed a three-factor structure analogous, although not identical, to that found in Study 1, the scores were calculated exactly as per Study 1, for consistency (Vaccine safety: α = 0.85; Diseases prevented: α = 0.83; Naturalistic views: α = 0.60). In the third section of the questionnaire, respondents completed the Profile of Emotional Competence (PEC) scale [20]. This scale measures people’s self-assessed intrapersonal and interpersonal emotional competence. It includes 50 items (e.g., “As my emotions arise, I don’t understand where they come from”, “If I wanted, I could easily influence other people’s emotions to achieve what I want”) to be answered on a 5-point scale from 1 (“it does not describe me at all”) to 5 (“it describes me very well). In the present study, we used both the intrapersonal (α = 0.72) and interpersonal (α = 0.76) subscales. Finally, the last section of the questionnaire asked respondents to answer a series of socio-demographic questions.

#### 3.1.3. Statistical Analyses

Descriptive statistical analyses were summarized producing frequency tables for categorical variables and using means with standard deviations (SDs) for continuous variables. Simple correlation between variables were computed. Parametric linear regression models were used to assess whether emotional competence predicted people’s scores on the three factors describing parents’ attitude towards vaccines. To test whether the mental image valence mediated participants’ score on “Vaccine safety”, we used the PROCESS macro, and we presented the results using betas for the standardized coefficients. Statistical significance was assumed at the 5% level. Statistical analyses were performed using SPSS version 20.0 statistical software (IBM Corporation).

### 3.2. Results

#### 3.2.1. Descriptive Statistics and Correlations

Compared to Study 1, the youngest child was on average 10 months younger, parents were more likely to hold a university degree, they had more diverse citizenships, and they were slightly wealthier (see Table 3). 

Participants reported a slightly positive mental image associated with vaccines and an intermediate level of both intrapersonal and interpersonal emotional competence (see Table 4). On average, parents showed some common fears and concerns about the side effects or adverse reactions following vaccination, many worries and concerns about the negative consequences that could be provoked by the diseases that are prevented by vaccines, and modest beliefs in natural protection and extreme views against vaccines.

When looking at the correlations, average mental images were positively related to intrapersonal emotional competence, whereas the correlation with interpersonal emotional competence was not significant. Further, average mental images were negatively correlated with scores of the “Vaccine safety” and “Naturalistic views” factors but positively correlated with the score of the “Disease prevented” factor. The same pattern of correlations was found for the relations between the intrapersonal and interpersonal emotional competencies and the three factors describing people’s attitude towards vaccinations (see Table 4).

#### 3.2.2. Mental Images

A first regression analysis tested the effect of the two emotional competence scales (intrapersonal and interpersonal) on the mean valence of the mental images reported by participants. Results showed a significant effect of intrapersonal emotional competence (*B* = 0.24, *SE* = 0.11, *t* = 2.20, *p* = 0.028, 95% C.I. (0.03, 0.45)), indicating that increasing intrapersonal competence led respondents to produce more positive mental images. In contrast, no significant effect of interpersonal emotional competence was found (*B* = −0.03, *SE* = 0.10, *t* = −0.27, *p* = 0.784, 95% C.I. (−0.23, 0.17)).

#### 3.2.3. Attitude towards Vaccines

A series of regression analyses were run to assess whether emotional competence predicted people’s scores on the three factors describing parents’ attitude towards vaccines. As for vaccine safety, the intrapersonal scale significantly predicted scores on this factor (*B* = −0.24, *SE* = 0.09, *t* = −2.62, *p* = 0.009, 95% C.I. (−0.42, −0.06)), whereas the interpersonal scale was not significant (*B* = 0.06, *SE* = 0.09, *t* = 0.63, *p* = 0.529, 95% C.I. (−0.12, 0.23)). Thus, the higher the intrapersonal emotional competence, the lower were parents’ concerns about vaccine safety. In a second regression analysis, the average mental image score was added to the model. Results showed that mental image had a significant effect in predicting scores on the factor describing concerns about vaccine safety (*B* = −0.33, *SE* = 0.03, *t* = −11.32, *p* < 0.001, 95% C.I. (−0.39, −0.27)), while the effects of intrapersonal (*p* = 0.650) and interpersonal (*p* = 0.760) emotional competence were not significant. This result indicates that the higher the mental images score the lower was participants’ tendency to worry about vaccine safety.

The same analyses were then repeated for the second factor describing worries and concerns about the negative consequences that could be provoked by the diseases that are prevented by vaccines. Results showed that the effects of intrapersonal and interpersonal emotional competence were not significant (respectively: *B* = 0.14, *SE* = 0.09, *t* = 1.45, *p* = 0.147, 95% C.I. (−0.05, 0.32) for the intrapersonal scale and *B* = 0.07, *SE* = 0.09, *t* = 0.79, *p* = 0.433, 95% C.I. (−0.10, 0.24) for the interpersonal scale). However, once the average mental image score was added to the model, it had a significant influence on parents’ worries about the diseases prevented by vaccines (*B* = 0.25, *SE* = 0.03, *t* = 7.90, *p* < 0.001, 95% C.I. (0.19, 0.31)). Therefore, increasingly positive mental images lead people to be more worried about the negative consequences that could be provoked by the diseases prevented by vaccines. Neither the intrapersonal (*p* = 0.840) nor the interpersonal (*p* = 0.206) emotional competence was significant.

Finally, a regression analysis was used to test the effect of emotional competence on the factor describing naturalistic views against vaccines. Results showed that neither scale had a significant effect (respectively: *B* = −0.04, *SE* = 0.07, *t* = −0.65, *p* = 0.516, 95% C.I. (−0.18, 0.09) for the intrapersonal scale and *B* = −0.12, *SE* = 0.06, *t* = −1.81, *p* = 0.070, 95% C.I. (−0.24, 0.01) for the interpersonal scale). Once the average mental image score was added to the model, it had a significant effect on naturalistic views against vaccines (*B* = −0.11, *SE* = 0.02, *t* = −5.02, *p* < 0.001, 95% C.I. (−0.15, −0.07)), indicating that increasingly positive mental images scores were associated with a lower tendency to hold naturalistic views against vaccines. Neither the intrapersonal (*p* = 0.849) nor the interpersonal (*p* = 0.151) emotional competence was significant.

#### 3.2.4. Mediation Analyses

**Figure 1:**
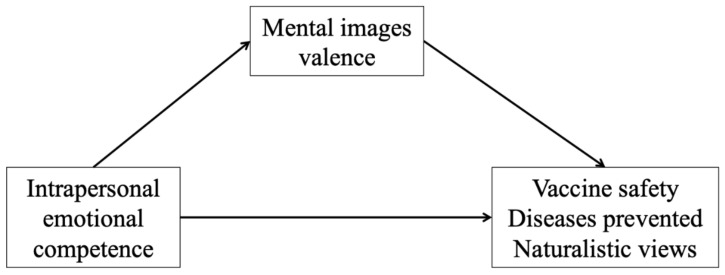
Path of the mediation analyses (Study 2).

When analyzing the mediation effect of mental images on the relation between intrapersonal emotional competence and participants’ score on vaccine safety, participants with a higher intrapersonal emotional competence were found to produce mental images with a more positive value than participants with lower emotional competence (*B* = 0.24, *p* = 0.028; Figure 1). In addition, more positive mental images corresponded to a lower score on the “Vaccine safety” factor (*B* = −0.33, *p* < 0.001). The indirect effect was significant (*B* = −0.08, *SE* = 0.04, *z* = −2.15, *p* = 0.032, 95% CI (−0.16, −0.01)). Similarly, for “Diseases prevented”, mental images predicted participants’ score (*B* = 0.25, *p* < 0.001), and the indirect effect was significant, too (*B* = 0.06, *SE* = 0.03, *z* = 2.10, *p* = 0.036, 95% CI (0.01, 0.14)). Finally, for “Naturalistic views”, positive mental images corresponded to a lower score (*B* = −0.11, *p* < 0.001) and the indirect effect was also significant (*B* = −0.03, *SE* = 0.01, *z* = −1.97, *p* = 0.049, 95% CI (−0.10, −0.01)). Reverse mediation models of interpersonal emotional competence as a mediator of the relationship between mental images and attitudes were also tested and yielded non-significant results.

### 3.3. Discussion

Having higher emotional competences was found to indirectly affect parents’ attitude towards vaccines. Indeed, parents with higher emotional competences concerning their own emotions (intrapersonal EC) were found to have more positive mental images associated with the word “vaccine”, and, in turn, to have more positive attitudes towards vaccines (i.e., lower concerns about side effects, higher concerns about the diseases prevented by vaccines, and lower beliefs in natural protection and less extreme views against vaccines). In other words, having high intrapersonal emotional competence implies a deeper recognition of the source of one’s own emotions, including fears and concerns about vaccines, and more functional and adaptive reactions to one’s feelings.

## 4. General Discussion

This paper confirms that temporal, partial, or complete vaccine refusal is only the tip of the iceberg. Indeed, the present work showed that psychological processes related to emotion and emotional competence have a significant impact on people’s attitudes towards vaccinations that, consistent with previous literature, shape decision making.

Study 1 demonstrated that attitudes towards vaccines can be characterized by three correlated but independent dimensions that are highly associated with vaccine refusal: common fears and concerns about adverse reactions (i.e., “Vaccine safety”), worries and concerns about the negative consequences of the diseases that are prevented by vaccines (i.e., “Disease prevented”), and beliefs in natural protection and extreme views against vaccines (i.e., “Naturalistic views”). Interesting results also emerge from the comparison between vaccine-refusing parents and parents who fully vaccinated their child on the average scores of the three factors measuring attitudes towards vaccines and from the correlational analysis. Specifically, hesitant parents have been shown to be more concerned with the safety of the vaccines than with the diseases that can be prevented, whereas parents who fully vaccinate seem to be considerably more worried about the diseases prevented by vaccines than by their safety. Moreover, although a consistent pattern of correlational analyses on the attitude scores was found for the whole sample, vaccine-refusing parents showed higher values compared to vaccine-accepting parents. It seems that, in comparison with parents who fully vaccinated their children, vaccine-refusing parents have a stronger negative association between the vaccine’s efficacy to prevent the diseases and both their beliefs about the safety of vaccines and their naturalistic views.

Study 2 demonstrated for the first time, as far as we know, a link between emotional competence and attitudes towards vaccines. In particular, parents’ intrapersonal emotional competences affected all dimensions of attitudes towards vaccines. Emotional competence has been previously found to be associated with several important outcomes, such as happiness, self-esteem, well-being, and life satisfaction [20]. In our study, being better able to identify, understand, express, regulate, and use one’s own emotions was a direct predictor of more positive spontaneous associations with the word “vaccine” and an indirect predictor of more positive attitudes towards vaccines. 

When thinking about vaccines, parents are likely to experience at least some level of worry or fear, even if they fully vaccinate (e.g., [7,30]). This emotional reaction is reasonable, considering that vaccines are nonetheless a medical intervention performed on healthy children (the status quo/frame of prevention is different from that of treatment), and that, even if the likelihood is extremely low, vaccines can cause adverse effects. Moreover, many myths about vaccines are circulating, especially on social media, and, unfortunately, searches online are prone to confirmation bias and false balance bias [38]. This is particularly relevant, as it has been shown that, in Italy, pediatricians are only the third most important source of information on vaccines for pregnant women, and that they are preceded by web sites and word of mouth [34]. Misinformation lingers in the memory [15,39], and information that has been encountered before is more likely to be believed as true (the so-called illusory truth effect) [40]. Likewise, correcting misconceptions based on misinformation is not an easy task [41] and can even backfire [42]. Cognitive-based individual differences do not seem to moderate these effects [40]. Our findings suggest that individual differences in emotional competence might enable people to make a final decision, despite being emotionally aroused. Having high intrapersonal emotional competence means being able to recognize that negative emotions arising from thinking about vaccines are often based on potentially inaccurate subjective perceptions rather than scientific facts. Therefore, we can conclude that higher (vs. lower) intrapersonal emotional competences lead people to produce more positive associations, in turn resulting in more favorable attitudes towards vaccines. 

Unexpectedly, interpersonal emotional competence was not found to play a role in parents’ vaccine attitudes. It is well known that vaccines do not only benefit directly the single individual being vaccinated, but also indirectly the community, by preventing the infection from spreading [43]. While the concept of herd (or community) immunity is complex, some evidence suggests that including an intervention stressing the pro-social benefits of vaccination by explaining community immunity can increase vaccination intentions [44,45], especially when the social benefit is stressed and vaccination is easy to obtain [46]. From this perspective, it could be expected that parents with higher interpersonal emotional competence would have a more favorable attitude towards vaccines and would be more motivated to vaccinate to protect susceptible community members. However, our data are not in line with this interpretation. One possible explanation is that parents were not aware of the reason why people are under vaccinated in their community. Indeed, a pro-social drive to vaccination is mostly effective when unvaccinated individuals have little control over their choice [47], but not when community members are susceptible as a result of their choice (or a choice on their behalf). In this case, they could be seen as “free-riders”, profiting from the immunity provided by other people, without risking themselves the extremely rare vaccine adverse events [48].

Another possible explanation is rooted in evolutionary psychology: parents are interested in their child’s survival and only appear to be motivated by pro-social appeals. For example, emphasizing societal benefits of vaccinations was found to have no effect on parents’ intention to vaccinate, whereas emphasizing the direct benefits to the child had a positive effect [49]. Additionally, it has been estimated that only between 1 and 6% of parents declare that benefit to others is their primary reason to decide to vaccinate their children, although many declare it is the second reason for doing it [50]. Whichever of the two hypotheses is true, should our findings be confirmed, intervention strategies, rather than stressing the pro-social benefits of vaccinating, should focus on aspects related to one’s own emotions.

Both studies have limitations. In Study 1, vaccination behavior was self-reported; thus, it is potentially affected by (more or less intentional) memory biases. Furthermore, the sample vaccine-refusal rate was lower than expected as per sample size calculation. Because of the small, but very consequential rate of vaccine-refusing parents in the population, Study 2 could only assess the effect of emotional competences on attitudes, not on self-reported behavior. However, as shown in Study 1, attitudes were strongly associated with self-reported behavior. Further, although we are aware that our studies are limited by using a non-validated measure of vaccine hesitancy, to our knowledge, no validated measure is available in Italian; however, the items were thoroughly developed in previous research and were previously published [27,35]. Finally, it should be noted that both studies were conducted in a peculiar context that has since changed significantly. At the time of our studies, no vaccine was mandatory in the Veneto Region (indeed it was the only Italian Region where the previous vaccine mandates were lifted in 2008), before the introduction of the current law (119/2017) mandating 10 vaccines for children up to 16 years, which has been introduced in 2017. Future studies should examine the role of emotional competences with the current vaccine policy and in other contexts with different or no mandates [32,51]. Additionally, it would be interesting to explore potential differences in mental images associated with the word “vaccine”.

Notwithstanding these limitations, the present work contributes in a significant way to extend the literature on vaccine hesitancy by adding to the explanatory model an affective component. These novel findings could be leveraged in future campaigns promoting vaccination. To this end, it is important to highlight that emotional competences are conceptualized as abilities that can be taught and learned [20]. Some findings suggest that healthcare professionals can improve their empathy in vaccination counselling sessions [52] and that dialogue-based interventions that embrace parents’ perspectives (such as motivational interviewing) are promising in addressing vaccine hesitancy and fostering vaccine uptake [53,54]. From this perspective, future research should explore the impact of increasing parents’ emotional competences. Another novelty of the present work is the demonstration that parents’ interpersonal emotional competences are not associated with their attitudes towards vaccines, thus suggesting why appeals to pro-social benefits of vaccination are often unsuccessful.

## Figures and Tables

**Table 1:** Participants’ characteristics (Study 1).

Variable	Category	F (%) or M (SD)
Child’s age	(Months)	46.55 (47.87)
Child’s gender	Male	1385 (50.6%)
	Female	1354 (49.4%)
Number of siblings	0	1282 (46.8%)
	1	1151 (42.0%)
	2 or more	306 (11.2%)
Mother’s age	(years)	38.72 (6.32)
Father’s age	(years)	41.86 (7.02)
Mother’s education	≤ Middle school	258 (9.5%)
	High school	1152 (42.1%)
	University	1328 (48.5%)
Father’s education	≤ Middle school	517 (18.9%)
	High school	1288 (47.0%)
	University	914 (33.4%)
Mother’s employment	Employed	2125 (76.5%)
	Not employed	649 (23.4%)
	Not present	4 (0.1%)
Father’s employment	Employed	2673 (96.2%)
	Not employed	71 (2.6%)
	Not present	34 (1.2%)
Mother’s citizenship	Italian	2581 (94.2%)
	Other	155 (5.7%)
Father’s citizenship	Italian	2584 (94.3%)
	Other	137 (5.0%)
Type of family	Couple	2610 (95.3%)
	Single parent	129 (4.7%)
Ease to get to theend of the end of the month	Very easily	397 (14.3%)
	Somewhat easily	1258 (45.3%)
	Somewhat difficult	912 (32.8%)
	Very difficult	211 (7.6%)
Who completed the questionnaire	Father	277 (10.1%)
	Mother	2462 (89.9%)

**Table 2:** Descriptive statistics and correlations (Study 1).

Parents Who Fully Vaccinated Their Child with no Voluntary Delays (*n* = 2512)				
	**1**	**2**	***M***	***SD***
1. Vaccine safety			2.35	0.74
2. Diseases prevented	−0.24 ***		4.70	0.45
3. Naturalistic views	0.52 ***	−0.22 ***	1.73	0.62
Vaccine-refusing parents (*n* = 227)				
	**1**	**2**	***M***	***SD***
1. Vaccine safety			3.90	0.82
2. Diseases prevented	−0.62 ***		3.26	1.11
3. Naturalistic views	0.58 ***	−0.64 ***	2.52	0.79

*** *p* < 0.001.

**Table 3:** Participants’ characteristics (Study 2).

Variable	Category	F (%) or M (SD)
Child’s age	(Months)	36.55 (18.43)
Child’s gender	Male	289 (48.7%)
	Female	298 (50.3%)
Number of siblings	0	257 (43.3%)
	1	246 (41.5%)
	2 or more	90 (15.2%)
Mother’s age	(years)	36.80 (5.53)
Father’s age	(years)	39.66 (6.69)
Mother’s education	≤ Middle school	47 (7.9%)
	High school	167 (28.2%)
	University	366 (61.72%)
Father’s education	≤ Middle school	76 (12.8%)
	High school	185 (31.2%)
	University	249 (42.0%)
Mother’s employment	Employed	466 (78.6%)
	Stay-at-home parent	60 (10.1%)
	Unemployed	54 (9.1%)
Father’s employment	Employed	487 (82.1%)
	Stay-at-home parent	2 (0.3%)
	Unemployed	25 (4.2%)
Mother’s citizenship	Italian	446 (75.2%)
	Other	137 (23.1%)
Father’s citizenship	Italian	420 (70.8%)
	Other	111 (18.7%)
Type of family	Couple	544 (91.7%)
	Single parent	39 (6.6%)
Difficulties to get to the end of the month	No difficulties	178 (30.0%)
	Few difficulties	159 (26.8%)
	Some difficulties	163 (27.5%)
	Many difficulties	44 (7.4%)
	Very many difficulties	32 (5.4%)
Who completed the questionnaire	Father	94 (15.9%)
	Mother	494 (83.3%)

**Table 4:** Descriptive statistics and correlations (Study 2).

	1	2	3	4	5	M	SD
1. Mental images						0.51	1.11
2. Intrapersonal EC	0.10 *					3.59	0.45
3. Interpersonal EC	−0.01	0.59 **				3.40	0.48
4. Vaccine safety	−0.45 ***	−0.11 **	−0.05			2.37	0.83
5. Diseases prevented	0.33 ***	0.10 *	0.08 *	−0.54 ***		4.33	0.83
6. Naturalistic views	−0.22 ***	−0.09 *	−0.11 **	0.58 ***	−0.42 ***	1.74	0.61

Note: Mental images are expressed as the average a cross the three images; * *p* < 0.05; ** *p* < 0.01; *** *p* < 0.001.

## Data Availability

The data presented in this study are available on request from the corresponding author. The data are not publicly available due to lack of permission to make them public. We are willing to provide any information (e.g., for meta-analytic purposes) in a timely manner to other researchers upon request.

## Supplementary Materials

The following are available online at https://www.mdpi.com/2076-393X/9/3/298/s1, Table S1. Attitude towards vaccines—Factorial analysis. Items English translation [and original Italian wording], and loadings from factorial analysis in Study 1. Table S2. Study 1—Regression analysis on vaccine-refusing behavior controlling for socio-demographic variables. Relationship between participants’ characteristics and vaccine-refusal in univariate analyses. Table S3. Study 1—Regression analysis on vaccine-refusing behavior controlling for socio-demographic variables. Full model predicting vaccine-refusal based on attitudes and socio-demographic variables.
