# Supplementary material for: The Role of Emotional Competences in Parents’ Vaccine Hesitancy
Source: Vaccines (Basel). 2021 Mar 22;9(3):298. doi: 10.3390/vaccines9030298 (PMC8005154; doi:10.3390/vaccines9030298)
Supplement: Supplementary file 1 [file vaccines-09-00298-s001.pdf]

Online supplementary material

Table S1. Attitude towards vaccines - Factorial analysis. Items English translation [and original Italian wording], and loadings from factorial analysis in Study 1.

**Factor 1 - Vaccine safety - common fears and concerns about vaccinations**	Loading
Healthcare professionals often refuse to recognize the link between vaccines and other illnesses like asthma or allergies. *[Gli operatori sanitari spesso non vogliono riconoscere il legame tra il vaccino e l'insorgere di diverse patologie (es., asma, allergie, altro).]*	.729
Not all adverse reactions to vaccines are recorded, and are therefore underestimated. *[Non tutte le reazioni avverse ai vaccini vengono registrate e quindi sono spesso sottovalutate]*.	.712
Too many vaccines are administered at once. *[Vengono fatte troppe vaccinazioni in un'unica soluzione.]*	.709
Professionals of the National Health System provide information only on the benefits of vaccination and not on the risks. *[Gli operatori del Sistema Sanitario Nazionale danno informazioni solo sui benefici delle vaccinazioni e non sui rischi.]*	.707
Serious and very serious side effects of vaccines are often kept hidden. *[Spesso gli effetti collaterali gravi o gravissimi dovuti al vaccino vengono tenuti nascosti.]*	.697
I am worried about the side effects that could occur immediately after the vaccination. *[Ho paura delle reazioni avverse subito dopo la vaccinazione.]*	.671
Vaccinations are administered when children are too young, they should be administered when children are older. *[Le vaccinazioni vengono effettuate su bambini troppo piccoli, bisognerebbe farle quando sono un po' più grandi.]*	.647
I am worried about possible serious side effects that can occur even a long time after vaccination. *[Temo che le reazioni avverse al vaccino possano presentarsi anche molto tempo dopo la vaccinazione.]*	.644
I would never forgive myself if my child was seriously damaged by vaccinations. [*Non mi perdonerei mai se mio figlio avesse gravi danni dalle vaccinazioni.*]	.611
Pediatric immunizations are above all a profitable economic business for pharmaceutical companies. *[I vaccini per l'infanzia sono soprattutto un business economico delle case farmaceutiche.]*	.604
In order to avoid serious side effects, vaccines should be individually tailored and not extensively administered to all children. *[Per evitare al massimo le reazioni avverse, le vaccinazioni dovrebbero essere decise caso per caso e non fatte in massa a tutti i bambini.]*	.569
Parents who refuse to vaccine their children are blamed by professionals from the National health system. *[Chi non vaccina viene colpevolizzato dagli operatori del Sistema Sanitario Nazionale.]*	.512
Professionals from the National Health System are competent and updated about vaccinations (reverse coded). *[Gli operatori del Sistema Sanitario Nazionale sono preparati ed aggiornati sulle vaccinazioni.]*	-.473
Professionals from the National Health System spend enough time to respond to any vaccine-related doubts or concerns (reverse coded). *[In genere gli operatori del Sistema Sanitario Nazionale sono disponibili ad affrontare dubbi o perplessità sulle vaccinazioni.]*	-.470
Whether or not to vaccinate their children is a private parents' decision and cannot be mandated. *[Vaccinare o meno i propri figli è una scelta privata che spetta esclusivamente ai genitori e non può essere imposta.]*	.440
I trust more health professionals outside the National health system. *[Ho più fiducia*	.439

nei consigli di sanitari al di fuori del Sistema Sanitario Nazionale].	
**Factor 2 - Diseases prevented - concerns about the diseases prevented by vaccines**	
It is important to vaccinate children because the prevented diseases could have very serious effects. *[È importante vaccinare i bambini perché le malattie che si prevengono possono avere effetti molto gravi.]*	.744
I am worried/concerned that my child could get some diseases if she/he is not vaccinated. *[Ho paura che il bambino contragga le malattie se non viene vaccinato.]*	.739
Children who attend nurseries and kindergartens need to be vaccinated, in order to avoid risks for other children. *[Per frequentare il nido e la scuola materna bisogna che i bambini siano vaccinati in modo da evitare rischi per gli altri bambini.]*	.714
If we stopped vaccinating, diseases that are now very rare could resurge. *[Se si smettesse di vaccinare molte malattie oggi rarissime potrebbero tornare in circolazione.]*	.690
I would never forgive myself if my child was seriously damaged by diseased that were preventable by vaccinating. *[Non mi perdonerei mai se mio figlio avesse gravi conseguenze da malattie che potevo prevenire con le vaccinazioni.]*	.618
**Factor 3 - Naturalistic views - Beliefs in natural protection and extreme views against vaccines**	
Disease prevented by a vaccine is less risky than vaccine itself. *[La malattia per la quale si vaccina è meno pericolosa del vaccino stesso.]*	.583
Breastfeeding protects your child against diseases and infections and it makes immunization unnecessary while he/she is breastfed. *[L'allattamento al seno protegge il bambino da qualsiasi infezione e quindi non è necessario vaccinarlo finché si allatta.]*	.521
Healthy lifestyles can prevent diseases and infections without the need to vaccinate the child. *[Seguendo stili di vita sani si possono evitare le malattie senza necessità di vaccinare il bambino.]*	.508
Air pollution is the most serious among risk factors that threaten health. *[L'inquinamento atmosferico è il più pericoloso tra i fattori di rischio per la salute.]*	.505
If vaccines are no longer mandatory, it means that they are no longer needed. *[Il fatto che le vaccinazioni non sono più obbligatorie significa che non sono più necessarie.]*	.426

Table S2. Study 1 - Regression analysis on vaccine-refusing behaviour controlling for socio-demographic variables. Relationship between participants' characteristics and vaccine-refusal in univariate analyses.

		OR [95% CI]	*p*
Child's age		1.00 [.99; 1.00]	.003
Child's gender	female vs. male (reference)	.96 [.73; 1.26]	.759
Number of siblings	1 vs. 0 (reference)	1.05 [.79; 1.41]	.725
	2 or more vs. 0 (reference)	1.11 [.71; 1.73]	.651
Mother's age		.98 [.96; 1.01]	.134
Father's age		.99 [.97; 1.01]	.310
Mother's education	high school vs. ≤ middle school (reference)	2.87 [1.38; 5.99]	.005
	university vs. ≤ middle school (reference)	3.16 [1.53; 6.55]	.002
Father's education	high school vs. ≤ middle school (reference)	1.25 [.84; 1.86]	.280
	university vs. ≤ middle school (reference)	1.51 [1.00; 2.28]	.048
Mother's employment	not employed vs. employed (reference)	.60 [.41; .86]	.006
Father's employment	not employed vs. employed (reference)	.50 [.16; 1.60]	.243
Mother's citizenship	Other vs. Italian (reference)	3.64 [1.34; 9.92]	.011
Father's citizenship	Other vs. Italian (reference)	1.97 [.91; 4.25]	.085
Type of family	single parent vs. couple (reference)	.72 [.35; 1.50]	.381
Ease to get to the end of the month	Very easily vs. Very difficult (reference)	1.63 [.87; 3.06]	.130
	Somewhat easily vs. Very difficult (reference)	1.31 [.74; 2.34]	.357
	Somewhat difficult vs. Very difficult (reference)	1.07 [.59; 1.95]	.816
Who completed the questionnaire	Father vs. Mother (reference)	1.84 [1.27; 2.69]	.001

Table S3. Study 1 - Regression analysis on vaccine-refusing behaviour controlling for socio-demographic variables. Full model predicting vaccine-refusal based on attitudes and socio-demographic variables.

	OR	[95% CI]	*p*
Vaccine safety	5.53	[4,05, 7.55]	< .001
Diseases prevented	.20	[.15, .26]	< .001
Naturalistic views	.72	[.51, 1.02]	.062
Child's age (≤ vs. > 6 years)	1.68	[.97, 2.92]	.065
Mother's education level (≥ high school vs. ≤ middle school)	3.64	[1.26, 10.48]	.017
Father's education level (≥ high school vs. ≤ middle school)	1.21	[.70, 2.09]	.502
Mother's citizenship (Italian vs. other)	6.25	[1,70, 22.96]	.006
Mother's employment status (employed vs. not)	2.10	[1,19, 3.70]	.010
Who completed the questionnaire (father vs. mother)	2.15	[1,13, 4.08]	.019
